# *In vitro* and *in vivo* evaluation of chlorhexidine salts as potential alternatives to potassium dichromate for *Eimeria maxima* M6 oocyst preservation

**DOI:** 10.3389/fvets.2023.1226298

**Published:** 2023-07-11

**Authors:** Lauren Laverty, Lesleigh C. Beer, Kristen Martin, Xochitl Hernandez-Velasco, Marco A. Juarez-Estrada, Marcela Arango-Cardona, Aaron J. Forga, Makenly E. Coles, Christine N. Vuong, Juan D. Latorre, Roberto Señas-Cuesta, Ileana Loeza, Latasha S. Gray, John R. Barta, Billy M. Hargis, Guillermo Tellez-Isaias, Brittany D. Graham

**Affiliations:** ^1^Department of Poultry Science, University of Arkansas, Fayetteville, AR, United States; ^2^Departamento de Medicina y Zootecnia de Aves, FMVZ, Universidad Nacional Autonoma de Mexico, Ciudad de México, Mexico; ^3^Department of Pathobiology, Ontario Veterinary College, University of Guelph, Guelph, ON, Canada

**Keywords:** coccidiosis, *Eimeria maxima*, chlorhexidine, autofluorescence, potassium dichromate

## Abstract

**Introduction:**

Coccidiosis caused by the *Eimeria* spp., an Apicomplexan protozoon, is a major intestinal disease that affects the poultry industry. Although most cases of coccidiosis are subclinical, *Eimeria* infections impair bird health and decrease overall performance, which can result in compromised welfare and major economic losses. Viable sporulated *Eimeria* oocysts are required for challenge studies and live coccidiosis vaccines. Potassium dichromate (PDC) is typically used as a preservative for these stocks during storage. Although effective and inexpensive, PDC is also toxic and carcinogenic. Chlorhexidine (CHX) salts may be a possible alternative, as this is a widely used disinfectant with less toxicity and no known carcinogenic associations

**Methods:**

*In vitro* testing of CHX gluconate and CHX digluconate exhibited comparable oocyst integrity and viability maintenance with equivalent bacteriostatic and bactericidal activity to PDC. Subsequent use of CHX gluconate or digluconate-preserved Eimeria oocysts, cold-stored at 4°C for 5 months, as the inoculum also resulted in similar oocyst shedding and recovery rates when compared to PDC-preserved oocysts.

**Results and discussion:**

These data show that using 0.20% CHX gluconate could be a suitable replacement for PDC. Additionally, autofluorescence was used as a method to evaluate oocyst viability. Administration of artificially aged oocysts exhibiting >99% autofluorescence from each preserved treatment resulted in no oocyst output for CHX salt groups.

## 1. Introduction

*Eimeria* is a genus of protozoan parasites that infect various animal species. The infection is prevalent in poultry, causing significant economic losses (1). The parasites cause intestinal coccidiosis, an extensive problem for the livestock industry that leads to severe economic losses. When shed from poultry in the feces, oocysts are unsporulated and, thereby, noninfective. The exogenous process in which oocysts mature in the environment is known as sporulation, an oxygen-intensive process. Only viable, sporulated oocysts will cause infection when ingested. Sporulation efficiency can be impacted by temperature, humidity, and oxygen availability (2). *Eimeria* oocyst degradation and sporulation assays are methods used to study the impact of compounds or conditions on integrity or exogenous development. However, they differ in their purpose and methodology. *Eimeria* oocyst degradation assays are used to determine the effectiveness of disinfectants or other treatments to damage the oocyst wall. In these assays, oocysts are treated with a chemical or physical agent, and then the percentage of degraded oocysts is determined by counting under a microscope. This assay aims to evaluate a treatment’s efficacy in killing or degrading *Eimeria* oocysts. On the other hand, *Eimeria* oocyst sporulation assays are used to study the exogenous life cycle of the parasite. For this, fresh, unsporulated oocysts are subjected to specific temperatures, humidity, and oxygen levels to promote their sporulation while being exposed to different chemicals or alternative treatments *in vitro* (3). The percentage of sporulated oocysts is then determined by counting under a microscope. This assay aims to understand the factors that influence the sporulation of *Eimeria* oocysts.

Potassium dichromate (PDC) has been used extensively as a reagent to promote *in vitro* sporulation and to preserve oocysts for prolonged periods (4). PDC (K_2_Cr_2_O_7_) is a strong oxidizing agent (5). PDC when used *in vitro* seems to promote the sporulation of Eimeria species oocysts, although the mechanism is not currently known (6). It is hypothesized that chromium ions may generate oxygen radicals in a manner similar to copper ions that promote sporulation (6, 7). The oxidizing agent also inhibits the growth of microorganisms, extending the viability of the oocysts (8). The purpose of PDC during *in vitro* sporulation is to provide a suitable environment and inhibit other oxygen-consuming microbes that may compete with the oocysts (3). PDC has several advantages as a reagent for storing *Eimeria* oocysts. It is easy to prepare, readily available, and inexpensive. The compound is stable and can be stored at room temperature for long periods without significant degradation (9). Furthermore, PDC has bactericidal and fungicidal properties to control microbial growth and preserve the oocysts’ integrity (7). Nevertheless, PDC has some limitations in its application for storing *Eimeria* oocysts. PDC is highly toxic and is considered a hazardous material (10). The substance has been categorized as a known carcinogen associated with various health problems, including lung cancer, skin sensitization, and liver and kidney damage (11). Environmental hazards related to PDC have also been of concern (12, 13). In addition, the oxidization may also alter the antigenicity of the oocysts, affecting the results of experiments that require functionally intact oocysts (14).

Alternative commercially available disinfectants to store *Eimeria* oocysts have been proposed to overcome the limitations of PDC. For example, sodium hypochlorite (NaClO) has been used to surface sterilize oocysts prior to long-term preservation in PDC. The compound is less toxic than PDC and is effective in inactivating microorganisms. However, extended periods of exposure to sodium hypochlorite negatively impacted sporulation and altered the integrity of the oocysts (15, 16). Although ethanol concentrations below 20% and formalin concentrations at 5% or less did not affect *E. tenella* sporulation, *in vivo* studies were not conducted to confirm viability after initial exposure and storage in ethanol or formalin treatments (16). Higher concentrations of formalin or ethanol completely obliterated the oocysts or significantly damaged the oocyst wall and negatively impacted sporulation. As a result, further studies evaluating alternatives to PDC are needed.

Chlorhexidine (CHX) salts are widely used as antiseptics and disinfectants in the healthcare industry (17). CHX is a cationic antiseptic and disinfectant exhibiting bacteriostatic, bactericidal, and anti-fungal properties depending on concentration (18, 19). CHX is typically available in gluconate, digluconate, or diacetate salt forms (20). CHX salts are available in different concentrations, with higher concentrations more effective at killing microorganisms (20). CHX salts are less toxic than PDC and have no known carcinogenic properties (21). The salts are also less environmentally hazardous and can be disposed of safely (22).

Since one of the challenges with studying *Eimeria* is maintaining viable oocysts for experimentation, a method to determine *Eimeria* oocyst viability without the need for a host would be valuable. The viability of oocysts declines depending on storage conditions and the age of the stocks (23). However, viable and nonviable sporulated oocysts can appear very similar morphologically. Previously, a high-fidelity fluorescent microscopy technique was used for *Eimeria* oocysts as a predictor of nonviability (24). When excited, the cytoplasm and sporocysts of morphologically normal but noninfective (nonviable) oocysts exhibit strong autofluorescence, whereas infective (viable) oocysts do not. Thus, autofluorescence can be used to indicate nonviability for *E. maxima* oocysts (25).

The objectives of the present study were to (1) assess the impact of CHX salts (CHX gluconate and CHX digluconate) on *E. maxima* oocyst sporulation and degradation *in vitro,* (2) evaluate *E. maxima* oocyst infectivity *in vivo* after long term storage in CHX salts, and (3) utilize fluorescent microscopy to determine oocyst viability *in vitro* in relation to *in vivo* infectivity.

## 2. Materials and methods

### 2.1. Challenge strain and stock

A pure stock of sporulated *Eimeria maxima* M6 (EMM6) oocysts was provided by Dr. John. R. Barta, University of Guelph, Canada. To obtain a sufficient number of oocysts for the experiment described, an *in vivo* amplification was required. Approximately 500 sporulated EMM6 oocysts/mL/chicken were administered to chickens between 9–10 days of age and reared in battery cages for the duration of the study. The birds were reared from day-of-hatch and not previously exposed to *Eimeria* oocysts. A low-dose challenge was selected to optimize oocyst production and shedding (26). Fecal samples containing unsporulated oocysts were collected from d5-8 post-challenge. Fecal material was homogenized and suspended in a sterile saturated salt solution, blended for 30–60 s, then sieved to remove coarse debris. The filtrate was centrifugated at 1,250 × *g* for 10 min to float oocysts away from fecal debris. Post-centrifugation, the supernatant containing oocysts was decanted, diluted in sterile distilled water (10x), and centrifugated again at 1,250 × *g* for 10 min to pellet the oocyst. The pellet of partially purified oocysts was resuspended in 2.0% or 2.5% potassium dichromate (w/v, aqueous) and transferred to Erlenmeyer flasks capped with sterile gauze at ~100,000 oocyst/mL to permit adequate aeration. The flask was placed on a rotary platform shaker operating at ~100 rpm at 26°C for 3–4 days or until microscopy confirmed sporulation. Following sporulation, the oocysts were resuspended in fresh 2.5% potassium dichromate and held at 4°C.

### 2.2. Chemicals

Chlorhexidine (CHX) salts evaluated in the present study included: CHX digluconate (Sigma, C9394), CHX diacetate salt hydrate (Sigma, C6143), and commercially available CHX gluconate (VETone brand). Aqueous CHX digluconate from a 20% stock solution was diluted in sterile H_2_O to obtain appropriate concentrations for the assays described below. Aqueous CHX gluconate from a 2% solution was also diluted in sterile H_2_O. A stock solution of CHX diacetate salt hydrate was made using ethanol and further diluted in sterile H_2_O. As a control, potassium dichromate (PDC) (Sigma, 207,802) was used at a final concentration of 2.5% (w/v).

### 2.3. *In vitro* assay 1

The purpose of *in vitro* assay 1 was to evaluate the antimicrobial efficacy of CHX salts when compared to PDC. Bacteria from poultry feces was selected to be used. A negative 0.9% saline control, a positive 2.50% PDC control, 2.0% CHX gluconate, and 20% aqueous CHX digluconate were used. Additionally, 0.14% CHX diacetate was used (data not shown). CHX diacetate, when used, exhibited notable antibacterial effects but rapidly degraded oocysts during incubation and was thus excluded from further trials. The 2.0% CHX gluconate was diluted to concentrations of 0.03, 0.05, 0.10, 0.20, and 0.40%. The 20% aqueous CHX digluconate was diluted at 0.125, 0.25, 0.50, 1.0, and 2.0% concentrations. These concentrations were selected to investigate the lowest efficacious dose of each solution. The solutions were diluted using a sterile 0.9% saline solution. A 10% fecal slurry was made by combining 100 g of fresh poultry feces from unchallenged birds and 900 mL of sterile, deionized H_2_O. The slurry was thoroughly mixed and allowed to separate at 1 g for 2 h. The resulting supernatant fluid was strained and kept for assay use. Before adding to the treatment solutions, the fecal slurry supernatant was serially diluted using 0.9% saline and drop-plated onto tryptic soy agar (TSA) plates (VWR, 880925) to quantify total aerobic bacteria present in the fecal slurry (7.13 Log_10_ CFUs/mL). For the assay, 4 mL of treatment solution and 1 mL of fecal slurry supernatant were combined into 15 mL conical tubes and incubated statically at 26°C (room temperature) for 116 h total. At 0 h, 21 h, 48 h, 75 h, and 116 h of incubation, each tube was line streaked onto a TSA plate using a sterilized inoculation loop. *N* = 5 replicate tubes and plates were used for each treatment dilution. Streaked plates were then incubated at 37°C aerobically for approximately 24 h before analysis. Plates with any bacterial growth present were considered positive. Data are shown in Table 1.

**Table 1:** Incidence of total aerobic bacteria recovered from a poultry-derived fecal slurry treated with potassium dichromate (PDC), chlorhexidine (CHX) gluconate, or CHX digluconate at five time points of evaluation (*in vitro* assay 1).

Treatment	Total aerobic bacterial recovery				
	0 h	21 h	48 h	75 h	116 h
Control saline solution	5/5 (100%)	5/5 (100%)	5/5 (100%)	5/5 (100%)	5/5 (100%)
2.50% PDC	5/5 (100%)	4/5 (80%)	4/5 (80%)	2/5 (40%) ^*,**^	0/5 (0%) ^*,**^
CHX gluconate (%)					
0.03	5/5 (100 %)	5/5 (100 %)	5/5 (100 %)	5/5 (100 %)	5/5 (100 %)
0.05	3/5 (60 %)	5/5 (100 %)	5/5 (100 %)	5/5 (100 %)	5/5 (100 %)
0.10	2/5 (40 %) ^*, **^	3/5 (60 %)	5/5 (100 %)	5/5 (100 %)	5/5 (100 %)
0.20	0/5 (0 %) ^**^	0/5 (0 %) ^**^	0/5 (0 %) ^**^	0/5 (0 %) ^**^	0/5 (0 %) ^**^
0.40	0/5 (0 %) ^**^	0/5 (0 %) ^**^	0/5 (0 %) ^**^	0/5 (0 %) ^**^	0/5 (0 %) ^**^
CHX digluconate (%)					
0.125	0/5 (0%) ^*,**^	4/5 (80%)	5/5 (100%)	5/5 (100%)	5/5 (100%)
0.25	0/5 (0 %) ^**^	0/5 (0 %) ^**^	0/5 (0 %) ^**^	1/5 (20 %) ^**^	0/5 (0 %) ^**^
0.50	0/5 (0 %) ^**^	0/5 (0 %) ^**^	0/5 (0 %) ^**^	0/5 (0 %) ^**^	0/5 (0 %) ^**^
1.0	0/5 (0 %) ^**^	0/5 (0 %) ^**^	0/5 (0 %) ^**^	0/5 (0 %) ^**^	0/5 (0 %) ^**^
2.0	0/5 (0 %) ^**^	0/5 (0 %) ^**^	0/5 (0 %) ^**^	0/5 (0 %) ^**^	0/5 (0 %) ^**^

Data are expressed as positive bacterial growth/total number of samples evaluated (%). ^*^Indicates significant differences within rows at *p* < 0.05. ^**^Indicates significant differences within columns between all treatment groups at *p* < 0.05.

### 2.4. *In vitro* assay 2

The purpose of *in vitro* assay 2 was to further assess the antimicrobial activity of CHX gluconate and CHX digluconate at their lowest efficacious concentration, 0.20, and 0.25%. Apart from these altered concentrations, the methods and materials are similar to the previously described *in vitro* assay 1. A negative 0.9% saline control, a positive 2.50% PDC control, CHX gluconate at 0.20% concentration, and CHX digluconate at 0.25% concentration were used. Treatment solutions were diluted to the desired concentration using sterile saline. Once again, the supernatant fluid from a 10% fecal slurry made from 100 g of fresh poultry feces and 900 mL of sterile, deionized H_2_O was used for the assay. The poultry feces and H_2_O were mixed thoroughly and allowed to separate at 1 g for 2 h, and the strained supernatant, containing 7.40 Log_10_ CFUs/mL, was kept for assay use. 4 mL of treatment solution and 1 mL of fecal slurry supernatant were combined in 15 mL conical tubes, thoroughly mixed, and statically incubated at 26°C for 50 h. Each tube was line streaked on TSA plates initially at 0 h and after 50 h incubation for bacteria recovery using a sterilized inoculation loop. For each treatment, there were *n* = 5 replicate tubes and plates. Streaked TSA plates were allowed to aerobically incubate at 37°C for approximately 24 h before analysis. Plates with bacterial growth present were considered positive. Data are shown in Table 2.

**Table 2:** Total aerobic bacterial recovery from poultry-derived fecal slurries treated with potassium dichromate (PDC), chlorhexidine (CHX) gluconate, or CHX digluconate at two time points of evaluation (*in vitro* assay 2).

Treatment	Total aerobic bacterial recovery	
	0 h	50 h
Control saline solution	5/5 (100 %)	5/5 (100 %)
2.50% PDC	5/5 (100 %)	3/5 (60 %)
0.20% CHX gluconate	0/5 (0 %) ^*^	0/5 (0 %) ^*^
0.25% CHX digluconate	0/5 (0 %) ^*^	0/5 (0 %) ^*^

Data expressed as positive bacterial growth/total number of samples evaluated (%). ^*^Indicates significant differences within columns at *p* < 0.05.

### 2.5. *In vitro* assay 3

The purpose of *in vitro* assay 3 was to determine whether CHX gluconate or digluconate would degrade oocysts during incubation at room temperature. A negative 0.9% saline control, a positive 2.50% PDC control, CHX gluconate at 0.20% concentration, and CHX digluconate at 0.20% concentration were selected to directly compare the two solutions. Treatments were diluted to the desired concentrations using sterile saline. As mentioned, CHX diacetate was also used but severely degraded the oocyst (data not shown). Each treatment was separated into 250 mL Erlenmeyer flasks and inoculated with approximately 75,000 oocysts/mL of sporulated EMM6 oocysts. The final volume was 20 mL. Oocyst dilution was completed using sterile saline. Flasks were then covered with sterile gauze pads and placed on a shaker plate to create gentle surface agitation in a 26°C incubator. The assay was allowed to incubate aerobically for 6d total, with 1 mL subsamples taken for enumeration at 0d, 24 h, 4d, and 6d. Oocysts were enumerated using the McMaster technique described by J.N. Hodgson (27) at a 1:100 dilution in a saturated salt solution, 600 μL/ chamber. McMaster slides containing samples were allowed to float undisturbed for 2–5 min before analysis. This method was repeated for each treatment at 0d, 24 h, 4d, and 6d. *N* = 3 replicate flasks per treatment. Data are shown in Table 3.

**Table 3:** Impact of potassium dichromate (PDC), chlorhexidine (CHX) gluconate, or CHX digluconate on total oocysts at 1d, 4d, and 6d of aeration at 26°C (*in vitro* assay 3).

Treatment	Total oocysts (log_10_ /mL)			
	Initial day 0	1 day aeration	4 days aeration	6 days aeration
Control saline solution	5.87 ± 0.011	5.77 ± 0.016	5.79 ± 0.052	5.66 ± 0.004
2.50% PDC	5.89 ± 0.015	4.82 ± 0.011	5.80 ± 0.023	5.65 ± 0.004
0.20% CHX gluconate	5.88 ± 0.020	5.81 ± 0.023	5.78 ± 0.020	5.60 ± 0.019
0.20% CHX digluconate	5.91 ± 0.006	5.79 ± 0.012	5.79 ± 0.028	5.62 ± 0.019

Data express as mean ± SE.

### 2.6. *In vitro* assay 4

The purpose of *in vitro* assay 4 was to further evaluate oocyst degradation during incubation in respective treatment solutions and calculate percent sporulation during this time. Fresh, unsporulated oocysts not exposed to PDC were collected for assay use. A negative 0.9% saline control, a positive 2.50% PDC control, CHX gluconate at 0.20% concentration, and CHX digluconate at 0.20% concentration were used. 250 mL Erlenmeyer flasks were used as vehicles. 15 mL of each treatment solution was inoculated with 5 mL of oocysts diluted in saline solution to achieve 10,000 oocysts/mL. Each flask was then covered with sterile gauze pads and placed on a shaker plate to achieve gentle surface agitation uniformly to oxygenate oocysts. The flasks were incubated aerobically at 26°C for 6d. Subsamples at a volume of 1 mL were collected at 0d, 4d, and 6d post-incubation to enumerate total oocysts, assess sporulation rate (%), and quantify viable aerobic bacteria for each treatment. Oocysts were enumerated using the previously described method at a 1:10 dilution. Percent sporulation was calculated by dividing sporulated oocysts by total oocysts (sporulated + unsporulated oocysts). Bacteria were quantified by serially diluting each solution using sterile saline and drop plating onto TSA plates. Inoculated plates were incubated aerobically at 37°C for 36 h before analysis. Data are shown in Table 4.

**Table 4:** *Eimeria maxima* M6 oocyst degradation, sporulation (%), and total aerobic bacterial recovery after 4 and 6d of incubation at 26°C and treatment with potassium dichromate (PDC), chlorhexidine (CHX) gluconate, or CHX digluconate solutions (*in vitro* assay 4).

Treatment	Total oocysts (log_10_)	Sporulation (%)	Bacterial recovered (log_10_ CFU/mL)
Initial day 0			
Control saline solution	5.70	0.00	6.52 ± 0.14^a^
2.50% PDC	5.70	0.00	0.00 ± 0.00^c^
0.20% CHX gluconate	5.70	0.00	0.00 ± 0.00^c^
0.20% CHX digluconate	5.70	0.00	0.67 ± 0.67^c^
4 days aeration			
Control saline solution	5.66 ± 0.003^ab^	90.0 ± 0.47^a^	8.13 ± 0.18^a^
2.50% PDC	5.70 ± 0.036^ab^	82.7 ± 1.87^b^	0.00 ± 0.00^c^
0.20% CHX gluconate	5.76 ± 0.032^a^	89.4 ± 1.23^ab^	0.00 ± 0.00^c^
0.20% CHX digluconate	5.62 ± 0.018^b^	88.4 ± 1.95^ab^	2.00 ± 0.00^b^
6 days aeration			
Control saline solution	5.71 ± 0.024	89.9 ± 1.12^ab^	8.30 ± 0.17^a^
2.50% PDC	5.75 ± 0.015	85.2 ± 1.54^b^	0.00 ± 0.00^b^
0.20% CHX gluconate	5.74 ± 0.037	92.7 ± 1.27^a^	3.33 ± 1.76^b^
0.20% CHX digluconate	5.67 ± 0.043	80.8 ± 0.46^b^	2.00 ± 0.00^b^

Data express as mean ± SE. ^a–c^ Values within the same column by day and parameter that do not share a common letter differ significantly (*p* < 0.05).

### 2.7. *In vivo* trial

**Figure 1:**
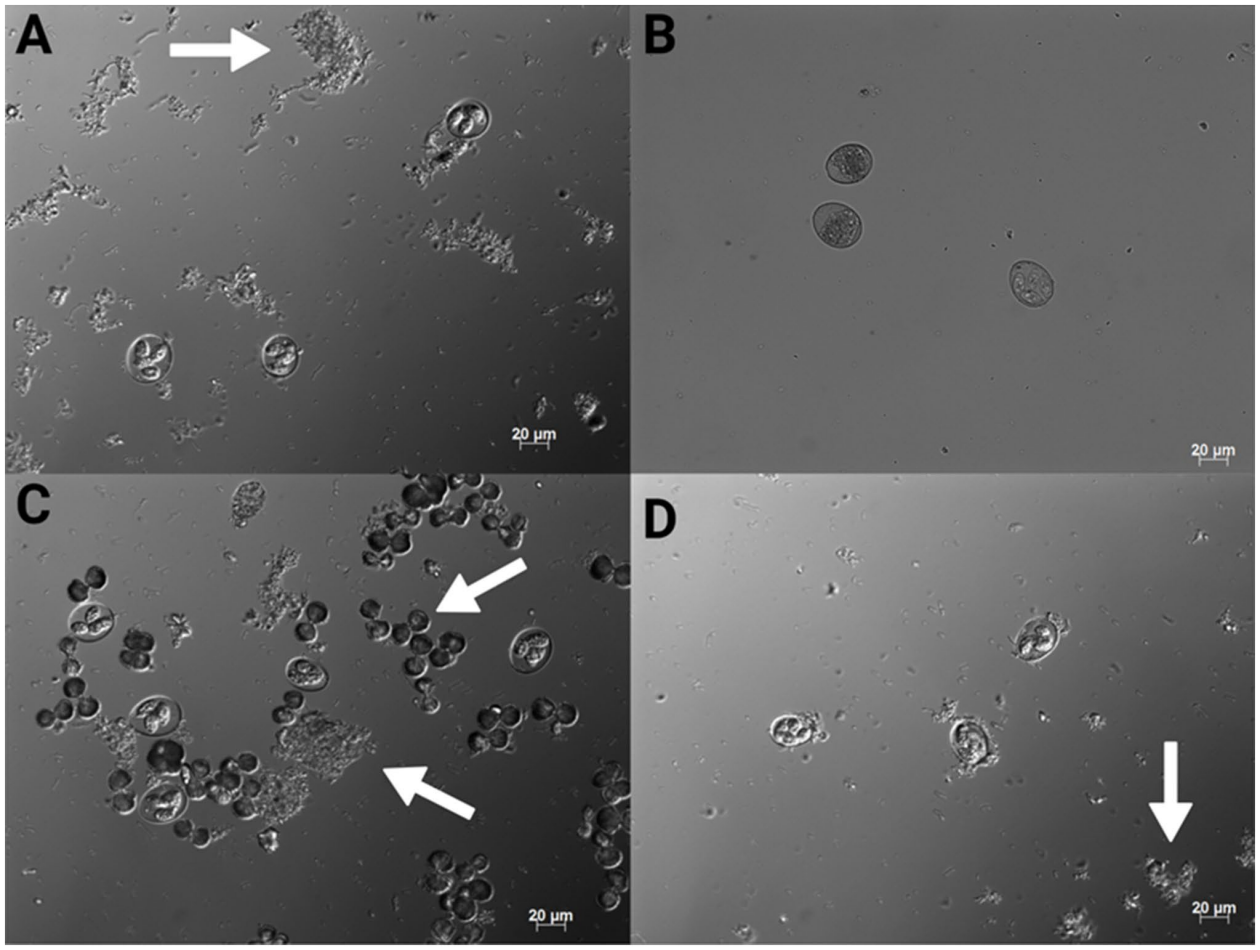
200x magnification conventional DIC photomicrographs of *Eimeria maxima* M6 stored in 0.9% saline **(A)**, 2.50% PDC **(B)**, CHX gluconate **(C)** and CHX digluconate **(D)**. The arrows indicate bacterial growth and also notable crystallization in **(C)** (Created with BioRender.com).

**Figure 2:**
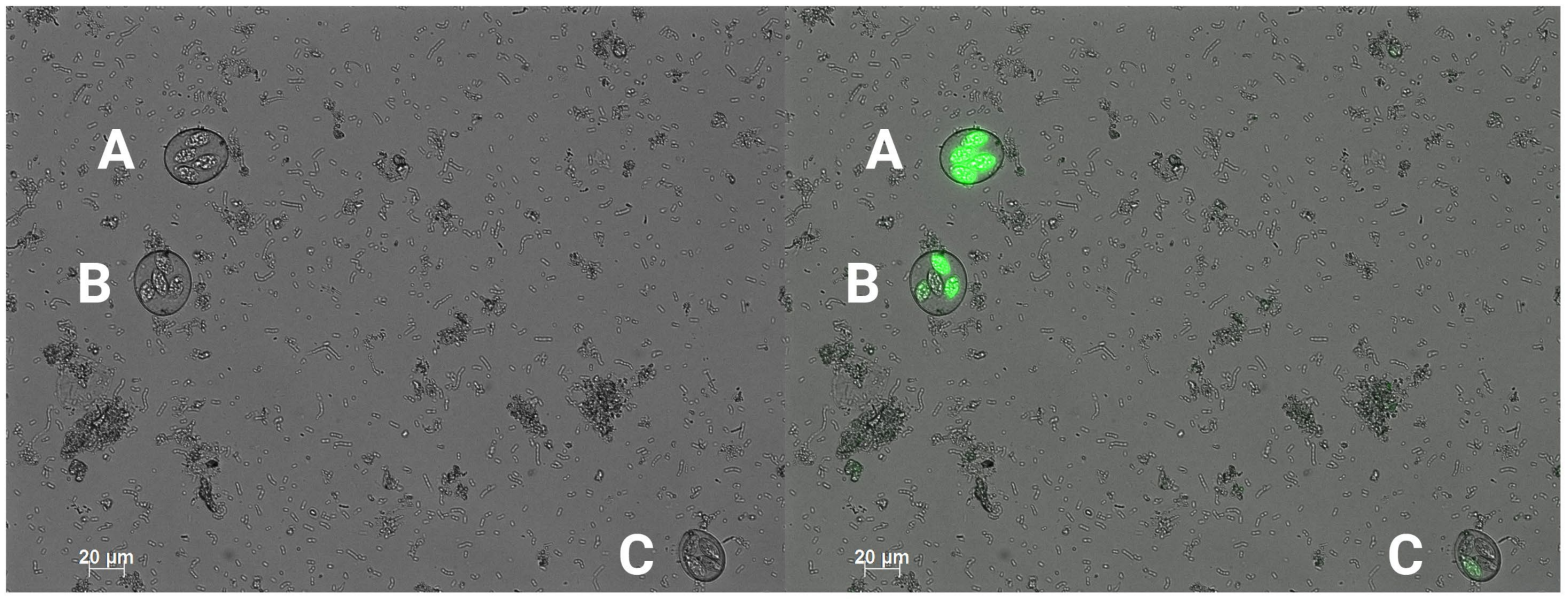
200x magnification conventional DIC photomicrograph (left) with a green fluorescent filter overlay (right). An example of a sporulated, fully autofluorescent oocyst (A), a sporulated, partially autofluorescent oocyst (B), and a sporulated, non-autofluorescent oocyst (C) (Created with BioRender.com).

**Figure 3:**
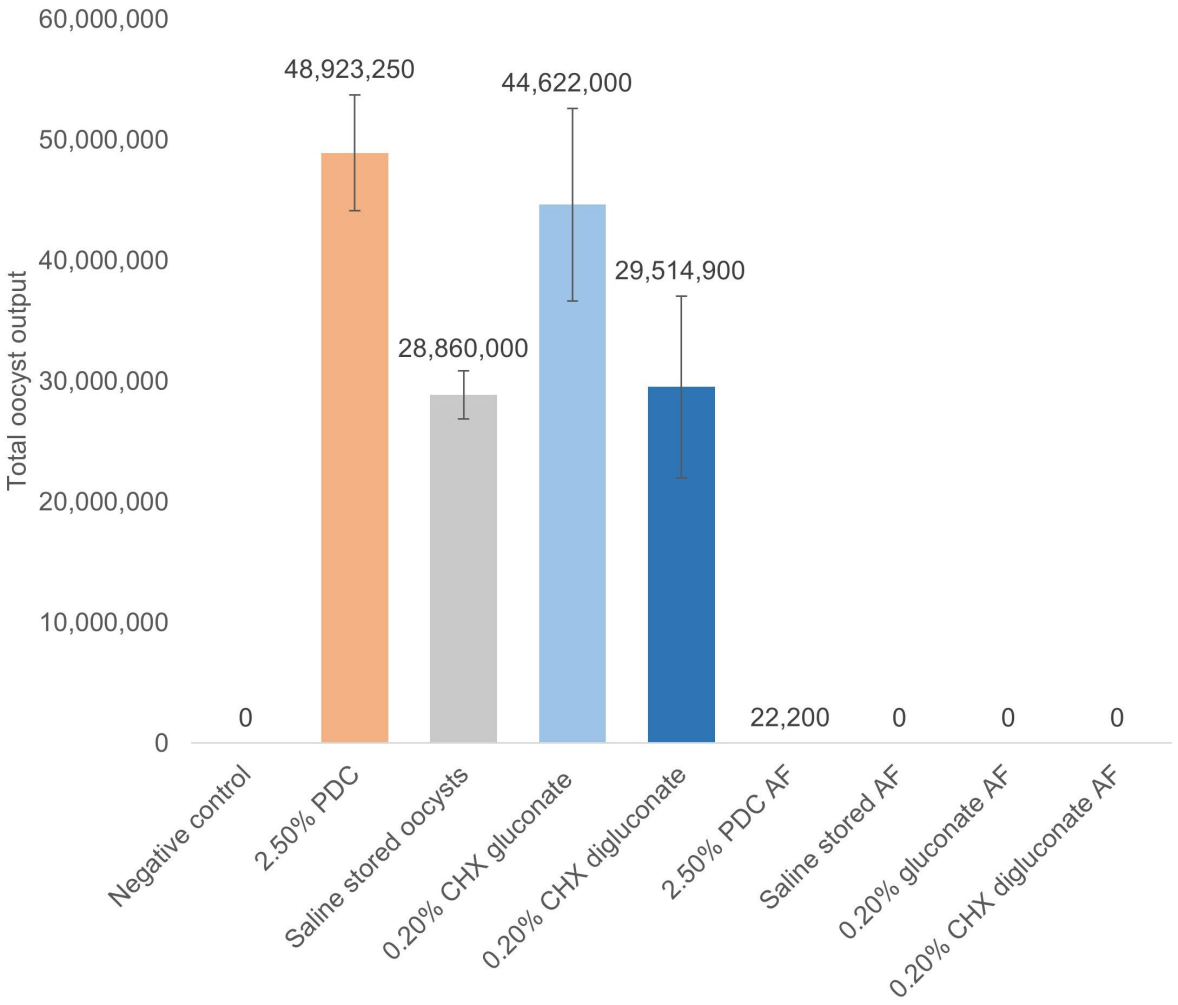
Total oocyst output from broilers challenged with non-autofluorescent or 100% autofluorescent (AF) EMM6 oocysts obtained from stocks stored for 5 months at 4°C in potassium dichromate (PDC), chlorhexidine (CHX) gluconate, or CHX digluconate from d5-9 post-challenge. *N* = 3 replicates/treatment group. Data express as mean ± SE. ^A−C^ Differing letters indicate significant differences between treatment groups at *p <* 0.05. Means further separated using Student’s *t* test. *In vivo trial*.

For the *in vivo* trial, freshly propagated sporulated EMM6 oocysts not previously exposed to PDC were placed into cold storage at 4°C for 5 months in respective treatments. Oocysts were sporulated in their respective treatment solutions under the same conditions specified in the Challenge strain and stock section. These treatments included a negative 0.9% saline control, a positive 2.50% PDC control, 0.20% CHX gluconate, and 0.20% CHX digluconate. EMM6 oocysts that had undergone *in vitro* sporulation were washed and diluted in the treatment solutions to a concentration of 10,000 oocysts/mL, 20 mL total, and stored for 5 months at 4°C. Once a week, the storage containers were opened and gently agitated to aerate oocyst stocks. After 5 months of storage, a 7 mL subsample of each treatment was removed and placed into an incubator at 45°C to accelerate the aging process and achieve 100% oocyst autofluorescence uniformly in 11 days. It has been demonstrated that oocysts stored at 45°C for 6 days would result in 93.3% autofluorescence (25). Additional time was added to ensure that 100% autofluorescence would be uniformly achieved. Autofluorescence was determined using the Axio microscopy software and fluorescent imaging at 488 nm/509 nm excitation/emission. Oocysts that exhibited vibrant green when excited were considered autofluorescent (Figures 1, 2). An EMM6 challenge for each treatment, including the 100% autofluorescent variant of each and the non-aged variant (<~10% autofluorescence), was prepared at a dose of 250 sporulated oocysts/mL, administered orally three times over 3 days for a total of 750 oocysts/bird at d9, d10 and d11 of age. This dose was selected to maximize output and avoid the crowding effect of oocysts (26). The challenge was accomplished by diluting washed oocysts with sterile saline in a 50 mL conical tube. Oocysts underwent two sterile, deionized H_2_O washes accomplished by centrifugation at 10 min at 845 g, discard of the supernatant fluid, and a final wash for the resulting oocyst pellet. Sterile saline was used to resuspend the oocyst pellet to the desired concentration. For the trial, 81 one-day-old male broiler chickens (Fayetteville, AR, United States) not previously exposed to *Eimeria* oocysts were randomly allocated to one of nine groups with three replicate cages per treatment (*n* = 3 chickens/ replicate). Chickens were placed in battery cages in a controlled, age-appropriate environment. Treatments consisted of Group (1) unchallenged, saline sham negative control, Group (2) challenged, 2.50% PDC-stored oocysts, Group (3) challenged, saline-stored oocysts, Group (4) challenged, 0.20% CHX gluconate-stored oocysts, Group (5) challenged, 0.20% CHX digluconate-stored oocysts, Group (6) challenged, 2.50% PDC-stored and 100% autofluorescent oocysts, Group (7) challenged, saline-stored and 100% autofluorescent oocysts, Group (8) challenged, 0.20% CHX gluconate-stored and 100% autofluorescent oocysts, and Group (9) challenged, 0.20% CHX digluconate-stored and 100% autofluorescent oocysts. Chicks received *ad libitum* access to water and feed for 23 days. All animal handling procedures complied with the Institutional Animal Care and Use Committee (IACUC) at the University of Arkansas, Fayetteville, under protocol #22007. Broilers were orally challenged with respective treatment (250 oocysts/1 mL/chicken) at d9. From d5-9 after the challenge administered on d9 of age, all feces excreted were collected into 4 L beakers containing 2.50% PDC. Collections were performed twice daily at 9:00 AM and 6:00 PM. For total oocyst output, evaluation was determined on a volumetric basis. Oocysts were enumerated using the McMaster technique, as previously discussed. Data are shown in Figure 3.

### 2.8. Data and statistical analysis

All data were subjected to ANOVA as a completely randomized design using the GLM procedure of SAS (28). Treatment means were partitioned using Tukey’s multiple range test at *p* < 0.05, indicating statistical significance.

Bacteria recovery from poultry fecal slurry positive samples was compared by a chi-square test of independence (29), testing all possible combinations to determine the significance (*p* < 0.05).

## 3. Results

The incidence of bacterial recovery from poultry-derived fecal slurries treated with PDC, CHX gluconate, or CHX digluconate at different concentrations and time points for *in vitro* assay 1 have been summarized in Table 1. The incidence of bacterial recovery from saline-treated samples was 100% at all time points evaluated. Samples treated with PDC had a significantly (*p* < 0.05) lower incidence of bacteria recovered at 75 and 116 h. The 0.10% CHX gluconate solution reduced bacterial recovery by 60% at 0 h. Nevertheless, CHX gluconate solutions at 0.20 and 0.40% inhibited bacterial growth at all points of evaluation (Table 1). The concentration of 0.125% CHX digluconate inhibited bacteria growth at 0 h. Interestingly, the rest of the higher concentrations of CHX digluconate inhibited bacteria growth at all evaluation points (Table 1). At 0 h, CHX gluconate at 0.10% or higher concentrations and all CHX digluconate concentrations significantly reduced bacterial recovery. Nevertheless, at 21, 48, 75, and 116 h of evaluation, 0.20 and 0.40% CHX gluconate and all concentrations of CHX digluconate (except the lowest, 0.125%) had similar results, with no bacteria being recovered. At 75 h recovery, 2.50% PDC reduced bacterial recovery to 40%, with 0% recovery at 116 h (Table 1).

Table 2 shows the incidence of bacterial recovery from poultry-derived fecal slurries treated with PDC, CHX gluconate, or CHX digluconate at two-time points for *in vitro* assay 2. Similar to *in vitro* assay 1, saline did not affect bacterial recovery at 0 h or 50 h, with bacterial recovery being 100% at both time points. There were no significant differences between PDC and the saline control. However, CHX gluconate at 0.20% and CHX digluconate at 0.25% inhibited bacterial recovery completely (Table 2).

Table 3 shows the effects of PDC, CHX gluconate, and CHX digluconate on EMM6 total oocyst concentration after 1d, 4d, or 6d of aeration *in vitro* assay 3. No significant differences in oocyst quantities were observed during the evaluation between groups (Table 3). However, oocysts evaluated in CHX digluconate exhibited poor oocyst wall integrity and were morphologically irregular compared to 2.50% PDC, saline, and CHX gluconate.

The effects of PDC, CHX gluconate, and CHX digluconate on EMM6 oocyst degradation, sporulation rate (%), and bacterial recovery after 4d and 6d of aeration for *in vitro* assay 4 have been presented in Table 4. At d4, a significant (*p* < 0.05) reduction in oocyst degradation was observed between CHX gluconate at 0.20% and CHX digluconate at 0.20% (Table 4). Sporulation (%) was significantly reduced with 2.50% PDC compared to the control saline solution. CHX digluconate at 0.25% significantly reduced bacterial recovery by 6.13 Log_10_ CFU/mL compared to the saline control. Furthermore, 2.50% PDC and 0.20% CHX gluconate completely inhibited bacterial growth (Table 4). At 6d, no significant differences were observed for oocyst degradation between all groups. Both 2.50% PDC and 0.20% CHX digluconate showed a significant reduction in sporulation (%) compared to CHX gluconate at 0.20%. Bacterial recovery was completely inhibited by 2.50% PDC, followed by CHX digluconate at 0.20% and CHX gluconate at 0.20% (Table 4).

The oocysts evaluated *in vitro* assay 4 were used for the inoculum in the *in vivo* study. Total oocyst output from broilers challenged with non-autofluorescent or 100% autofluorescent EMM6 oocysts obtained from stocks stored for 5 months at 4°C in 2.50% PDC, 0.20% CHX gluconate or 0.20% CHX digluconate has been presented in Figure 3. Total oocyst output was highest for chickens that received EMM6 oocysts stored for 5 months in 2.50% PDC, followed by CHX gluconate at 0.20%. There were no significant differences in total oocyst output post-challenge with the autofluorescent oocysts. Although not significant, there were oocysts recovered from two of the three replicates for the group that received the 100% autofluorescent oocysts stored in 2.50% PDC (Figure 3). This output could be due to sample contamination during handling and analysis, but additional studies to confirm these findings would be beneficial.

## 4. Discussion

*Eimeria* spp. oocysts have been traditionally stored in potassium dichromate (PDC), a compound that effectively eliminates microorganisms (4). However, toxic and carcinogenic properties of PDC have been described highlighting the need for a safer alternative for preserving *Eimeria* spp. oocysts (30). In the healthcare industry, chlorhexidine (CHX) salts are widely used as antiseptics and disinfectants. Disruption of membrane potential rather than ATPase inactivation is the primary method by which CHX is lethal to cells (31). CHX has been shown to have concentration-dependent antimicrobial effects on *E. coli* (32). The investigators demonstrated that the membrane integrity of *E. coli* was shown to decrease following treatment with 0.03 mmol/L of CHX. The internal cellular structure is damaged, possibly due to coagulation following biocidal CHX exposure suggesting that CHX could be used as a potential alternative to PDC.

In the present study, two independent *in vitro* fecal slurry assays demonstrated that 0.20% CHX gluconate or 0.25% CHX digluconate possessed greater antimicrobial activity than 2.50% PDC. Both 0.20% CHX gluconate and 0.25% CHX digluconate were more efficacious at each time point evaluated. By 116 h, there was no viable bacteria recovered from both CHX treatments and the 2.50% PDC control. At each time of analysis prior to 116 h, incidence of recovery was 0% for both 0.20% CHX gluconate and 0.25% CHX dicluconate apart from a 20% incidence at 75 h for 0.25% CHX digluconate. This demonstrates that CHX salts could not only successfully reduce the load at these concentrations, but also do so in a shorter period of time when compared to 2.50% PDC. On the other hand, while bacteria recovery after 4d of aeration at 26°C when EMM6 oocysts were treated with 2.50% PDC or 0.20% CHX gluconate were completely inhibited, 0.20% CHX digluconate was limited to a 6.13 log reduction of bacteria compared to the control saline solution. This could indicate a difference in potency between CHX gluconate and CHX digluconate when used at the same concentration. Bacteria were not recovered at 6d of aeration from EMM6 oocysts stored in 2.50% PDC. Nevertheless, CHX gluconate or digluconate solutions showed a significant reduction in bacteria at 4.97 and 6.30 logs, respectively, compared to the control saline solution. None of the treatments impacted oocyst wall integrity after 1, 4, or 6d of incubation at 26°C or negatively impacted sporulation rates at 4 or 6 days of incubation compared to the PDC control in the present study. This indicates that CHX gluconate or digluconate could be used instead of PDC for *in vitro* sporulation of EMM6 oocysts, as they are both as effective, if not more effective, as PDC at reducing bacterial incidence and promoting sporulation *in vitro.*

PDC has been a reliable reagent for storing *Eimeria* oocysts for prolonged periods. Remarkably, 0.20% CHX gluconate was statistically comparable to 2.50% PDC in preserving oocyst infectivity during 5 months of *in vitro* storage at 4°C based on total oocyst output. Storage of EMM6 oocysts in 0.20% CHX digluconate markedly reduced total oocyst output compared to 2.50% PDC control and numerically lowered output compared to storage in 0.20% gluconate. This difference in oocyst output between CHX gluconate and CHX digluconate suggests that the CHX salt form can impact the viability of the oocysts during storage. Furthermore, these results show that CHX gluconate appears to be a better CHX salt for preserving EMM6 oocysts than CHX digluconate. The differences between the two salt forms and their effect on oocysts during long term cold storage should be evaluated. Although CHX diacetate results were not shown in the present manuscript, CHX diacetate degraded EMM6 oocysts and detrimentally affected sporulation of the remaining oocysts. Additionally, the pH did not appear to be a factor throughout the assay. This requires further investigation to better understand the mechanistic differences between the acetic acid and gluconic acid components of CHX salt forms. Perhaps the diacetate acts more readily on the oocyst wall components causing irreversible damage. CHX diacetate degraded EMM6 oocysts. The use of CHX diacetate for preserving oocysts should be avoided or investigated further.

As previously published (25), autofluorescence is a powerful tool in assessing the viability of *Eimeria* oocysts. Detectable autofluorescence in artificially aged oocysts further validates this method as an indicator for oocyst nonviability based on downstream total oocyst output. To effectively control and treat coccidiosis, it is essential to accurately determine the viability of *Eimeria* oocysts. A recent study in our laboratory showed that autofluorescence can distinguish viable from nonviable *Eimeria maxima* oocysts (25). Nonviable oocysts exhibit a strong autofluorescence signal, while viable oocysts exhibit a weak or absent signal. One of the key advantages of using autofluorescence to assess oocyst viability is that it is non-invasive and non-destructive. This means that oocysts can be assessed without being damaged, which is important when working with limited numbers of oocysts or when trying to maintain the integrity of oocyst populations for further study. This technique has the potential to greatly enhance our understanding of the biology and epidemiology of *Eimeria* and could help to improve the control and treatment of coccidiosis in a wide range of animal species (33, 34).

In general, the ideal preservation method should maintain oocysts’ viability, infectivity, and antigenicity while minimizing toxicity and environmental impact. In summary, the results of this study suggest that 0.20% CHX gluconate is a promising and reliable alternative to PDC for storing *Eimeria* oocysts. CHX gluconate at 0.20% concentration was effective not only at killing bacteria, but also maintained oocyst wall integrity and infectivity while in storage at 4°C over 5 months. Furthermore, CHX salts are less toxic than PDC and have no known carcinogenic properties. The CHX salts are also less environmentally hazardous and can be disposed of safely. This manuscript successfully demonstrated that CHX salts, specifically CHX gluconate, could be a viable alternative to PDC for storing oocysts. However, further research is needed to determine whether CHX salts can alter the antigenicity of the oocysts. Overall, using CHX salts for storing *Eimeria* oocysts should be considered a safer and more environmentally friendly alternative to PDC.

## Data availability statement

The original contributions presented in the study are included in the article/supplementary material, further inquiries can be directed to the corresponding author.

## Ethics statement

The animal study was reviewed and approved the Institutional Animal Care and Use Committee (IACUC) at the University of Arkansas, Fayetteville under protocol #22007.

## Author contributions

JB, BH, GT-I, and BG conceptualized the study. LL, LB, KM, and MA-C handled the methodology. AF, MC, and CV oversaw the software. JB, GT-I, and BG validated the study. RS-C, IL, LG, and JL performed the formal analysis. BG and GT-I conducted the investigation. LL, BG, and GT-I prepared and wrote the original draft. XH-V, MJ-E, and GT-I contributed to review and editing of the manuscript. GT-I and BH were in charge of the project administration and funding acquisition. All authors contributed to the article and approved the submitted version.

## Funding

This project was funded by USDA Animal Health Awards (FY2021 & FY2022), and by USDA-NIFA Sustainable Agriculture Systems, Grant No. 2019-69012-29905. Title of Project: Empowering U.S. Broiler Production for Transformation and Sustainability USDA-NIFA.

## Conflict of interest

The authors declare that the research was conducted in the absence of any commercial or financial relationships that could be construed as a potential conflict of interest.

## Publisher’s note

All claims expressed in this article are solely those of the authors and do not necessarily represent those of their affiliated organizations, or those of the publisher, the editors and the reviewers. Any product that may be evaluated in this article, or claim that may be made by its manufacturer, is not guaranteed or endorsed by the publisher.
